# Influence of Adding a Small Quantity of Rose Quartz on the Thermal Stability and Adhesive Properties of Silicone Pressure-Sensitive Adhesives

**DOI:** 10.3390/ma19091865

**Published:** 2026-05-01

**Authors:** Adrian Krzysztof Antosik, Marcin Bartkowiak

**Affiliations:** Department of Organic Chemical Technology and Polymer Materials, Faculty of Chemical Technology and Engineering, West Pomeranian University of Technology in Szczecin, Piastów Ave. 42, 71-065 Szczecin, Poland

**Keywords:** silicone PSA, rose quartz, thermal resistance, silicone self-adhesive tapes, power engineering

## Abstract

Silicone pressure-sensitive adhesives are a prominent group of adhesive materials used in many contemporary industrial sectors. This is due to their high resistance to difficult operating conditions, especially high temperatures. They are used, among other areas, in the automotive industry or in power engineering, as fastening or insulation systems operating at high temperatures. Previous studies have demonstrated the beneficial effect of mineral fillers on further increases in thermal resistance and dimensional stability of silicone pressure-sensitive adhesives. This paper presents the results of research on the effect of adding rose quartz as a filler to silicone pressure-sensitive adhesives based on polydimethylsiloxanes, on the adhesion parameters of the obtained adhesives and their thermal resistance and dimensional stability at elevated temperatures. The self-adhesive tapes obtained showed increased resistance and thermal stability while maintaining the required performance parameters. Among the tested compositions, optimal PSA parameters were achieved for Q2-7358 resin filled with 0.5 pph of rose quartz particles: adhesion exceeded industrial requirements by more than 15%, and tack met those requirements. Furthermore, low (and consistent) shrinkage (0.4% after one week) and cohesion—evaluated as hold time > 72 h—were recorded. As the most important parameter for studied compositions, thermal resistance (SAFT) substantially increased (>225 h) in comparison to neat resin (150 h).

## 1. Introduction

Adhesives have become an essential component in a wide range of industries, providing unique properties that address diverse application needs. These substances can be categorized into various groups based on several key factors, including the origin of the raw materials used in their production, their physical properties such as adhesion, cohesion, and shrinkage, and their specific bonding mechanisms, whether chemical or physical. Furthermore, they are distinguished by their application and curing techniques, which may involve thermal processes, UV-curing, or the use of ready-made compositions. A primary distinction frequently utilized in the industry is the classification of adhesives into liquid or semi-liquid types and pressure-sensitive adhesives, commonly known as PSAs [1,2,3,4].

Pressure-sensitive adhesives have integrated deeply into numerous industrial sectors, providing application-friendly alternatives to traditional fastening systems like rivets. These materials are characterized as “everlasting” adhesives because they maintain their adhesive integrity throughout their entire period of use. Unlike conventional adhesives, PSAs can be applied to surfaces without forming permanent chemical bonds, allowing for relatively easy removal—a feature that sets them apart from more rigid bonding agents [1,5,6,7]. Among the various types of PSAs available, silicone pressure-sensitive adhesives (Si-PSAs) have attracted increased scientific and industrial interest over the last two decades.

Silicone pressure-sensitive adhesives (PSAs) are frequently utilized in labels and self-adhesive tapes. They possess several key properties, including strong adhesion to low-surface-energy substrates and remarkable stability in harsh conditions, such as high humidity, UV radiation, or chemical exposure [8,9]. These adhesives are composed of a resin and a silanol-terminated silicone polymer dissolved in a hydrocarbon solvent like xylene or toluene [10]. Although numerous reports on various PSA formulations can be found in the literature, silicone-based adhesives remain in the minority despite their exceptional properties. Naturally, the cost of these adhesives is a relevant factor, but it is offset by their suitability for specialized applications. For instance, Lee and his colleagues [11] examined the adhesion of platinum-catalyzed silicone PSAs. Their findings indicated that both tack and peel strength increased with a higher concentration of platinum. Other research has demonstrated that silicone PSAs can endure the high temperatures and severe conditions often found in modern technology [12]. In a different set of experiments, a group of researchers explored the thermal stability of silicone adhesives, observing that silicone polymers, such as polydimethylsiloxane, undergo thermal degradation in the presence of air at elevated temperatures [13].

The application of nanofillers in various types of pressure-sensitive adhesives (PSAs) was an inevitable consequence of research into diverse fillers utilized in the plastics industry. Following the development of composites and nanocomposites, studies began to investigate the impact of minor inorganic admixtures on the mechanical and adhesive properties of PSAs. Kostyuk A.V. et al. demonstrated the influence of various solid fillers on the properties of isobutylene-based PSAs [14,15,16,17]. Similar adhesives modified with white carbon black (WCB) were studied by Kang et al. [18]. Czech et al. examined the effect of silica nanoparticles on the properties of polyacrylate PSAs [19,20]. Furthermore, the modification of polyacrylate PSAs with montmorillonite (MMT) nanoclay was investigated by Kajtna et al. [21] and Oh et al. [22]. Silicone adhesives have also been modified by Zhang et al., among others, using silica and alumina nanofillers [23].

Rose quartz represents one of the most distinctive polymorphic varieties of silicon dioxide (SiO_2_), distinguished by its unique pink coloration of varying saturation. Unlike crystalline varieties such as amethyst, where color results from color centers associated with iron ion impurities, the coloration of massive rose quartz is most commonly attributed to microscopic inclusions of fibrous borosilicate minerals, structurally similar to dumortierite. From a crystallochemical perspective, this mineral is characterized by a trigonal crystal system and a hardness of 7 on the Mohs scale [24]. Spectroscopic studies indicate that the subtle variations in transparency and hue are governed by light scattering on submicroscopic foreign phase inclusions and the presence of trace amounts of titanium, manganese, or phosphorus within the crystal lattice [25,26,27,28].

The primary objective of the present study was the modification of a selected silicone pressure-sensitive adhesive to improve its long-term thermal resistance. Although Si-PSAs are characterized by high thermal stability, the integration of mineral fillers offers a pathway to further enhancement. Building on prior research, which demonstrated that such additives not only stabilize the adhesive layer but also provide fire-retardant benefits, rose quartz was employed as a modifying agent in this work [29,30,31,32,33]. Extensive analysis was conducted to determine the filler’s effect on the tapes’ functional properties, thermal endurance, and the shrinkage behavior of the modified Si-PSA compositions.

## 2. Materials and Methods

### 2.1. Materials

The following materials were used in the studies:

- Silicone adhesive resin DOWSIL™ 7358 (Q2-7358) from Dow Corning (Midland, MI, USA).
- Silicone adhesive resin DOWSIL™ 7388 (Q2-7388) from Dow Corning (Midland, MI, USA).
- Silicone adhesive resin DOWSIL™ 288 Mica Binder (288) from Dow Corning (Midland, MI, USA).
- NOVIPER DB 50—Bis(2,4-dichlorobenzoyl) peroxide (DClBPO) from Novichem (Chorzów, Poland)—cross-linking agent.
- Toluene from Carl Roth (Karlsruhe, Germany)—solvent.
- Rose quartz from Poppystones (Sztutowo, Poland).

Rose quartz for the studies was ground into dust using a professional laboratory grinder, working at a speed of 28,000 rpm, with small portions (40–50 g) of mineral ground for 10 min per portion. The obtained filler was then analyzed for the particle size distribution using a laser diffraction analyzer Partica LA-950V2 (Horiba, Kyoto, Japan). The results of the analysis are presented in Table 1 as the mean value of 8 samples with standard deviation: the equivalent diameters of filler particles (D_0.5_, D_0.9_), surface weighted mean D_3.2_, and volume-weighted mean D_4.3_.

The particle size distribution of the rose quartz sample was analyzed using laser diffraction. The material reached a stable dispersion state, yielding a median particle size D_0.5_ of 8.39 µm. The distribution is characterized by a moderate right-skewness, as indicated by a volume-weighted mean D_4.3_ of 19.44 µm and a D_0.9_ value of 31.25 µm. These parameters are consistent with a fine-grained mineral powder after mechanical processing.

Silicone PSA resins employed in this study are commercially available products with the following composition:

- Q2-7358: Polydimethylsiloxane gum and resin diluted with a xylene and toluene mixture. Silicone solids content ca. 56.5%;
- Q2-7388: Polydimethylsiloxane gum and resin diluted with xylene. Silicone solids content ca. 56.5%;
- 288-MB: Polydimethylsiloxane polymer and resin dispersed in xylene. Silicone solid content ca. 61%.

### 2.2. Preparation of One-Sided Self-Adhesive Tape

**Figure 1:**
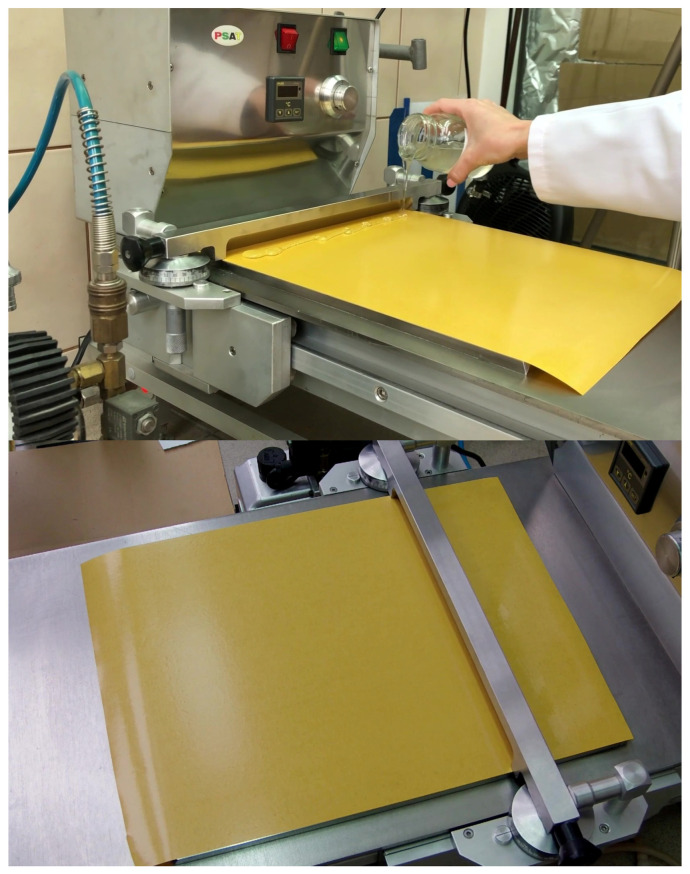
Laboratory doctor blade coating machine.

Selected silicone resins (Q2-7358, Q2-7388, and 288) were mixed with toluene and an appropriate amount of DClBPO (1.5 pph based on polymer solid content). Next, rose quartz was added to the samples in the amounts of 0.1, 0.5, 1.0, and 3.0 pph (parts per 100 parts of resin), and mixed thoroughly to obtain a homogeneous composition. The compositions were then coated onto a 50 µm polyester film, using the laboratory blade coater presented in Figure 1. The adhesive films were then dried at 120 °C for 10 min. After the crosslinking process, the adhesive films were protected with a second layer of fluorosilicone-polyester film (Dolpap Ltd., Chojnów, Poland) and cut into strips for the application tests. Coating weight was evaluated using a circular sample cutter for specimen collection and an analytical balance, and the obtained tape samples achieved a dry coating weight of 45 g/m^2^.

### 2.3. Pot Life

Pot life is defined as the stability of a prepared pressure-sensitive adhesive composition during storage. It refers to the time when the viscosity of silicone resin increases (2–4 times compared to the viscosity of the initial composition). As a result, the increase in viscosity makes it impossible to apply the adhesive to the carrier film.

The evaluation of pot life was performed immediately after mixing the composition and was carried out at room temperature, by the use of the rotational viscometer IKA Rotavisc me-vi (IKA-Werke GmbH & Co. KG, Staufen im Breisgau, Germany). Three samples were prepared for each composition, and their viscosity changes were evaluated. The results represent the mean of the three replicates, with the sample standard deviation reported.

### 2.4. Peel Adhesion

**Figure 2:**
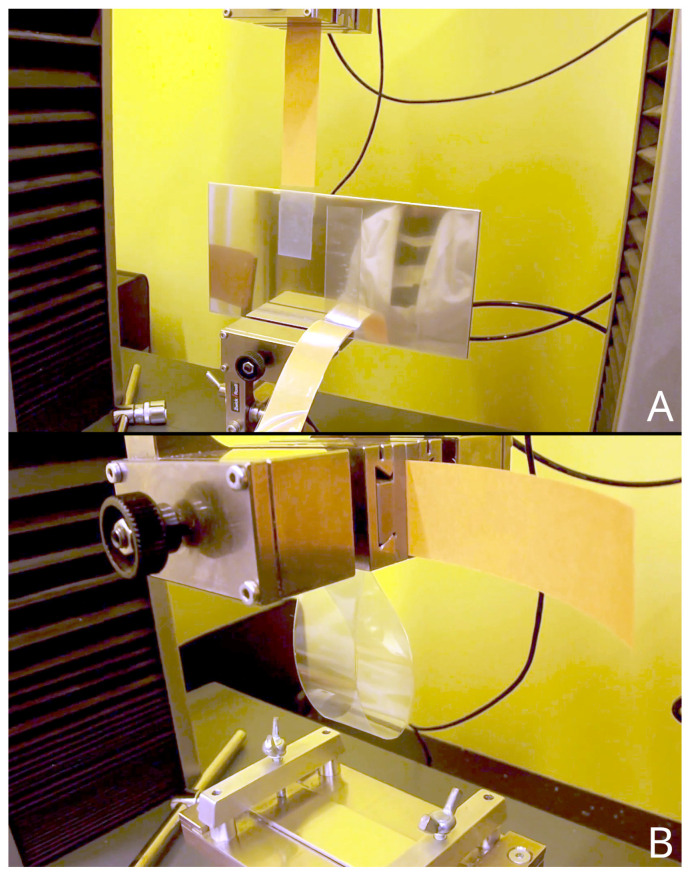
Peel adhesion (A) and tack tests (B) according to FINAT industrial standards.

Adhesion is the phenomenon of mutual attraction at the interface between two bodies or phases. This phenomenon is critical in bonding processes and is governed by interfacial forces. In this study, adhesion strength was measured according to the industrial standard developed by Fédération Internationale des Fabricants et Transformateurs d’adhesifs et thermocollants sur papiers et autres support (FINAT) FTM 1—“Peel adhesion (180°) at 300 mm per minute” [34]. The test procedure is as follows. Samples of PSA tapes, in the form of strips 25 mm wide and at least 175 mm long, were partially adhered to a test surface (stainless steel) using a 2 kg roller. After 20 min, the test plate and the free end of the strip were placed in the grips of a testing machine so that the peel angle was 180 degrees. The machine was set to a separation rate of 300 mm/min. At least three strips should be tested from each material sample, and at least several measurement points from the middle area of the sample should be included in the average peel force value. A tensile testing machine, Zwick/Roell Z-25 (Zwick/Roell GmbH & Co. KG, Ulm, Germany), was used to measure the force required to peel off the tape from the steel at a specified angle and with a defined speed for the presented studies. The sample during the test is presented in Figure 2A.

### 2.5. Tack

Tack refers to an adhesive’s ability to form an instant bond with a surface upon brief contact, without the application of pressure. It can also be defined as the force required to separate two surfaces after a very short contact time. Measurements were conducted in accordance with the FINAT FTM 9—“Loop tack measurement” standard [34]. Samples consisting of PSA-coated strips (25 × 175 mm) were formed into loops with the adhesive side facing out and secured in the upper grips of a tensile tester. A horizontal steel plate (25 × 30 mm) served as the contact surface. The loop was lowered until a 25 × 25 mm area contacted the plate, followed by immediate separation at a constant speed of 300 mm/min to measure the peak force of separation. At least five strips should be taken from each sample of PSA material to evaluate the average value of the separation force. The sample during the test is presented in Figure 2B.

### 2.6. Cohesion

**Figure 3:**
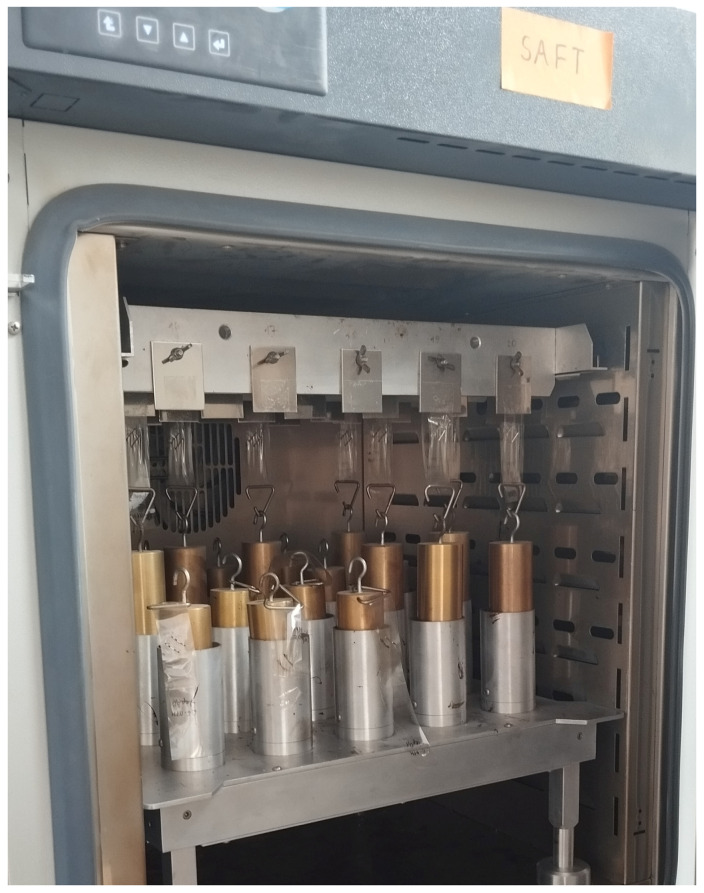
Shear strength test according to FTM 8 industrial standard.

Cohesion is the intermolecular attraction within a phase. Cohesive strength is the internal strength and integrity of an adhesive bond. Along with adhesion, it is one of the most important properties of pressure-sensitive adhesives and tapes, and is also found as a shear strength of PSA [7]. Its value is influenced by factors such as test temperature, concentration, type of cross-linking compound, and the thickness of the adhesive film. Cohesion was determined according to the standard FINAT FTM 8—“Resistance of shear from a standard surface” [34] at both room temperature and an elevated temperature of 70 °C. A strip of PSA-coated material was attached to the steel plate with a contact area of 25 × 25 mm, using a standard 2 kg roller. The plate with the attached tape was suspended from a rack placed in the thermostatic chamber. The free end of the PSA tape strip was loaded with a hanging weight of 1 kg after an appropriate time (not less than 5 min and not more than 10 min after rolling), as presented in Figure 3. The time was measured until the tape sample was separated from the plate by a sheared joint. The measurements were carried out at 23 °C and 70 °C. Moreover, the samples were conditioned for at least 4 h at room temperature before evaluation. Measurements were conducted simultaneously for four samples of each composition, utilizing the available slot capacity of the thermostatic chamber. Results falling below the industrial minimum threshold are presented as the mean value of the measurements.

### 2.7. Thermal Resistance

To determine thermal resistance, a SAFT (Shear Adhesion Failure Temperature) test was performed. Samples were prepared using the same procedure as for the cohesion tests. A 1 kg mass was attached to each sample placed in the conditioning chamber (Figure 3). The temperature was then slowly raised from 23 °C to 225 °C at a constant heating rate of 1 °C/min. The temperature at which the bond failed was recorded, along with the time of separation and the nature of the adhesive joint failure. The tests were carried out with four samples for each composition, and the mean value of temperature resistance was calculated.

### 2.8. Shrinkage

The shrinkage test was carried out according to the FINAT FTM 14—“Dimensional stability” standard [34]. A PVC film with a self-adhesive layer, in the form of a rectangle 10 cm × 10 cm, was applied to an aluminum plate. A vertical and horizontal incision, each 8 cm long, was made in the center of the film to form a cross. The prepared sample was then aged in an oven at 70 °C. Shrinkage was examined with a special magnifying glass after 10 and 30 min; 1, 3, 8, and 24 h; and 2, 3, 4, 5, 6, and 7 days. The width of the slits created by the cuts was measured to determine the extent of shrinkage. Results represent the mean value of 8 points of measurement recorded for each sample.

### 2.9. DSC

The crosslinking process of the composition has a measurable thermal effect, which can be tested using the differential scanning calorimetry method. DSC Apparatus Q100 by TA Instruments (New Castle, DE, USA) was used during the studies. The measurements were carried out under a nitrogen atmosphere, and the samples were scanned from −100 to 300 °C at a heating rate of 5 °C/min. Each measurement was repeated 3 times for different samples, and mean values and SD for n = 3 were calculated.

### 2.10. Simulation Studies on Different Surfaces

For the optimal composition—which achieved the highest performance metrics such as adhesion, cohesion, tack, shrinkage, and thermal resistance on a steel substrate—analogous performance tests were conducted on various other substrates. These included Copper, Aluminum, Brass, Glass, Polyethylene (PE), Polyacetal (POM), Polyamide (PA), Polyethylene terephthalate (PET), High-Impact Polystyrene (HIPS), Acrylic glass (PMMA), Polyvinyl chloride (PVC), Acrylonitrile butadiene styrene (ABS), and Polycarbonate (PC).

## 3. Results and Discussion

### 3.1. Pot Life Evaluation

**Figure 4:**
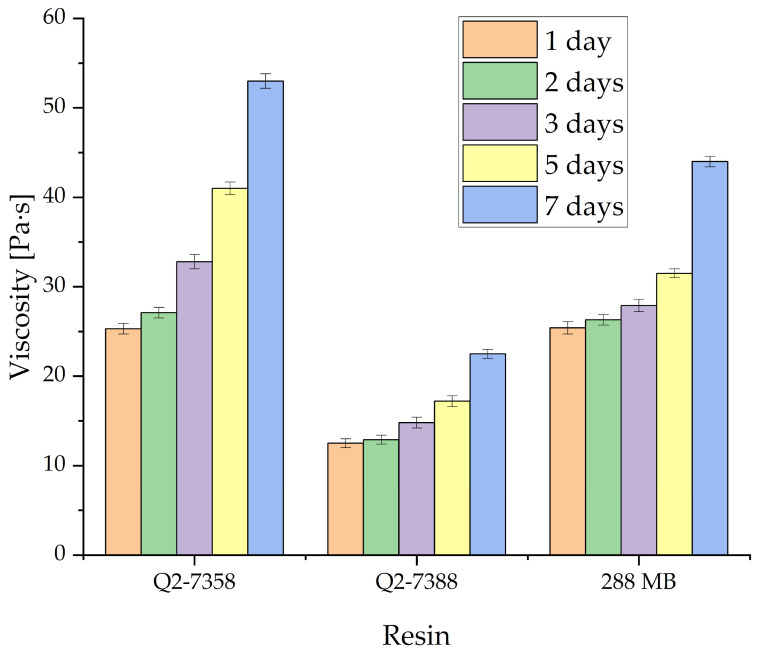
The viscosity of various silicone pressure-sensitive adhesives with the addition of 3 pph rose quartz.

The influence of rose quartz addition on the change in viscosity over time for three tested Si-PSA resins is detailed in Table 2 and Figure 4. Measurements were conducted for samples with a peak filler concentration of 3 pph due to the influence of filler addition on the viscosity of the initial composition, typical for the addition of a solid filler to a liquid matrix. For each series, the increase in viscosity after 7 days was almost twofold, with the fastest increase for Q2-7358. Small changes were observed up to 48 h for Q2-7358, and up to 72 h for Q2-7388 and 288 MB. After that, the changes accelerated, and after 9–10 days, all compositions gelled. The observed changes in viscosity of the tested samples are similar to comparable systems containing silicon resins and other silica fillers [29,35,36,37]. A comparison of viscosity changes between the resin–filler–crosslinker system and the filler-free system reveals a more rapid increase in viscosity over time, although the overall growth trend remains similar. The accelerated viscosity increase following the addition of quartz filler is a noteworthy phenomenon, particularly given the very low filler concentrations employed. This reduction in pot life may be attributed to strong physical interactions between the silica surface and the polymer chains. The presence of native surface silanol groups promotes the formation of an immobilized interfacial layer, where polymer mobility is substantially restricted. These filler-resin interactions, combined with the high surface area of the particles, may facilitate the formation of a transient three-dimensional network (physical gelation). This leads to an earlier macroscopic rise in viscosity and a shortened pot life [38,39].

### 3.2. Application Properties of Si-PSA with Rose Quartz

**Figure 5:**
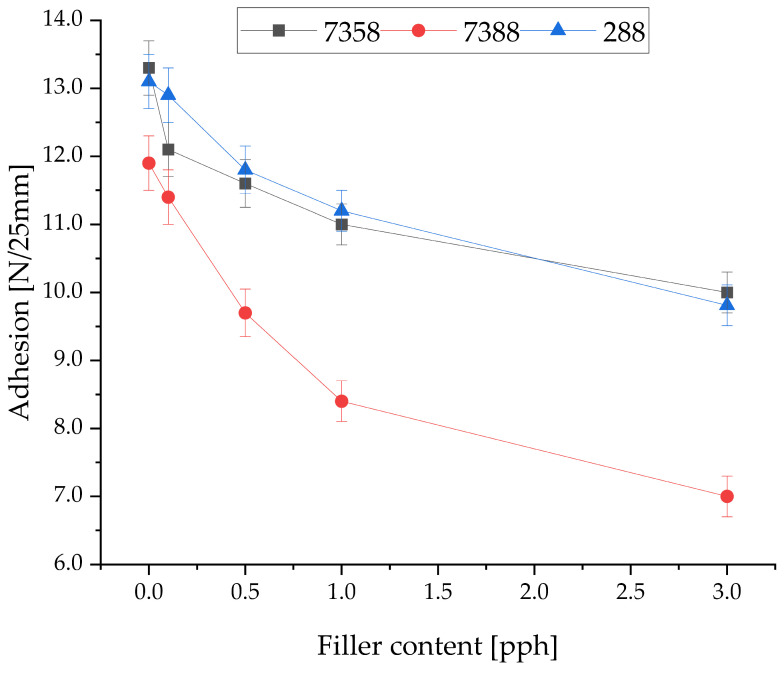
Adhesion of various silicone pressure-sensitive adhesives with the addition of rose quartz.

**Figure 6:**
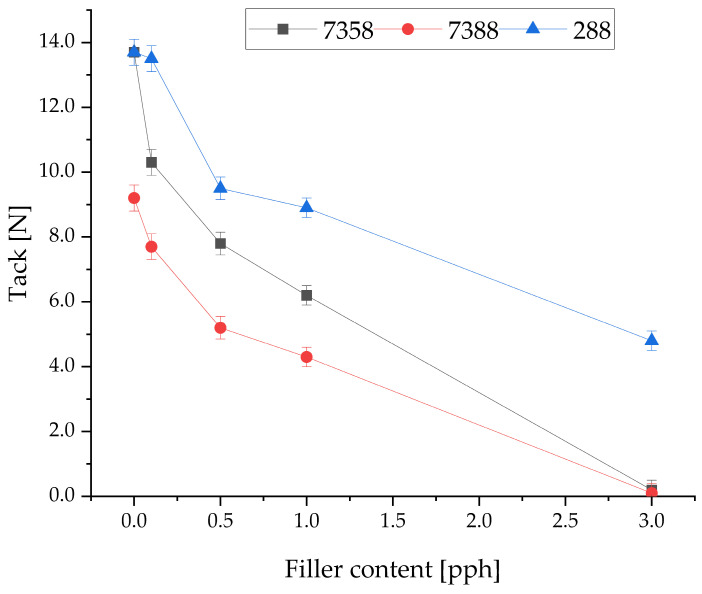
Tack of various silicone pressure-sensitive adhesives with the addition of rose quartz.

The influence of filler concentration on peel adhesion and tack of Si-PSA-RQ compositions is presented in Figure 5 and Figure 6, respectively. The addition of filler for each PSA resin results in an evident decrease in both properties. It can be concluded that such a phenomenon is commonly observed in similar adhesive–filler systems [40,41]. However, fillers have an important impact on thermal stability, which was concluded in previous studies, cited previously in the Introduction. Thus, the concentration of the filler (whichever one) cannot be increased too much. It is important to consider that acceptable limits of peel adhesion and tack for industrial purposes are 10 N/25 mm and 8 N, respectively. According to this, Q2-7388 with the addition of rose quartz is unsuitable for industrial application under the tested conditions, due to the rapid loss of adhesion and tack, even with the smallest amounts of filler used. For Q2-7358 and 288-MB, the observed changes in adhesion and tack are still acceptable, and over the threshold of industrial usability, for a lower concentration of the filler. Si-PSA resin 288-MB results were most promising for further tests, closely followed by the results of resin Q2-7358. It is particularly noticeable that the tack decreases most rapidly for these two resins. The rose quartz concentration level above 0.5 pph decreases tack below the acceptable value.

Next, the most important feature of pressure-sensitive adhesives is cohesion, also described as shear strength. Cohesion can be defined as the internal consistency of the adhesive layer and its durability. This property is strongly related to temperature conditions; cohesion evaluation is carried out both at room temperature and at an increased temperature (70 °C). An additional important test is the SAFT test, which means Shear Adhesion Failure Temperature. It evaluates the thermal resistance of the adhesive layer to shear forces.

The results of cohesion and SAFT evaluation are presented in Table 3.

It is necessary to mention here the threshold values resulting from industrial requirements. The cohesion value should be above 72 h of sample hold time (according to FTM-8). A SAFT value above 225 °C means very good thermal resistance of the adhesive layer. As can be seen in Table 3, cohesion at room temperature was sufficient for every tested composition. Increasing the temperature to 70 °C indicated the weak points of the tested samples in relation to the filler concentration. Si-resins Q2 (both) kept their shear strength filled with rose quartz up to 0.5 pph. Filler addition closer to 1 pph and above led to a notable decline in cohesion. Resin 288-MB had the worst cohesion results at 70 °C, even with the lowest rose quartz concentration (~8 h). It should be noted that changes in the amount of rose quartz caused an initial increase in cohesion and then a sharp decrease. This is similar to Q2 resins for RQ concentrations >1 pph, although this could be explained by the weakening of the adhesive structure by the filler. For 288-MB resin, it seems more complicated, because the initial cohesion is about 10 times lower than required, for 0.1 pph of filler. Next, cohesion increases along with the filler concentration increase, and with a 3 pph slope down near 2 h only. A similar phenomenon was observed while working with a different resin–quartz filler composition. We may hypothesize that for the 288-MB resin, there is an optimal filler loading that effectively enhances the interfacial/internal cohesion of the adhesive layer. This is attributed to the optimal dispersion of the filler. Higher concentrations, however, may lead to the formation of local agglomerates, which act as stress concentrators and weaken the adhesive layer.

The SAFT test for all resin-RQ compositions results in a decrease in thermal resistance as the response for the filler concentration increases. This is not an exceptional phenomenon, as we observed similar results with some silica fillers studied in our previous work.

The shrinkage test results, obtained according to the industrial test, are shown in Table 4.

Shrinkage evaluation proved that the addition of mineral filler to Si-PSA resin results in the self-adhesive layers, which were dimensionally stable for a prolonged time at 70 °C. Of course, shrinkage increased over the course of a week for each sample, and the observed changes were almost linear up to 5 days, after which the shrinkage value stabilized. However, with increasing filler concentration, shrinkage decreased by up to half for both Q2-resins. Anyway, all results are in the range of industrial acceptance limits (below 0.5% for most PSAs) and even below the limit called excellent dimensional stability (0.3%).

### 3.3. DSC Analysis

**Figure 7:**
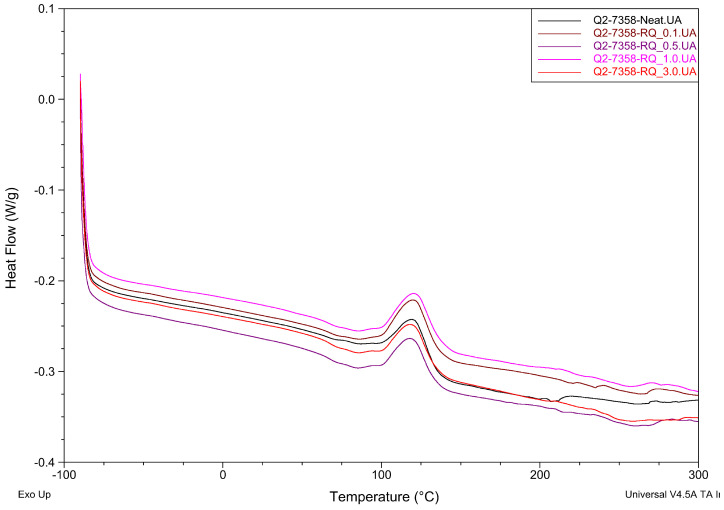
The effect of rose quartz concentration on the maximum temperature and thermal effect of the cross-linking process of Q2-7358-RQ adhesive films.

**Figure 8:**
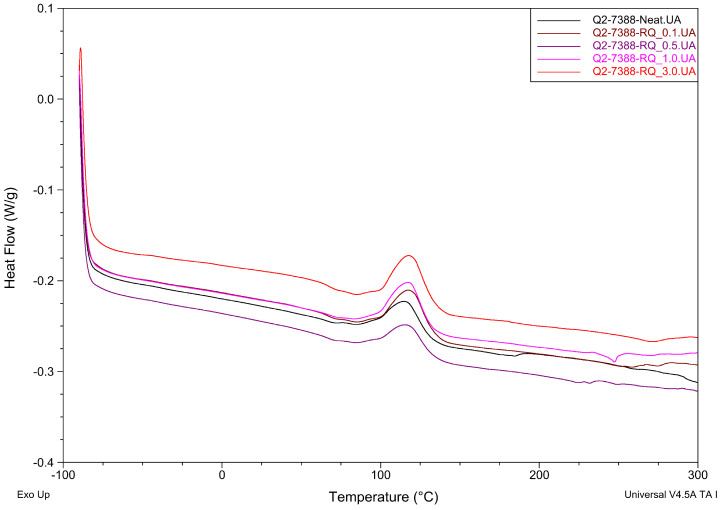
The effect of rose quartz concentration on the maximum temperature and thermal effect of the cross-linking process of Q2-7388-RQ adhesive films.

**Figure 9:**
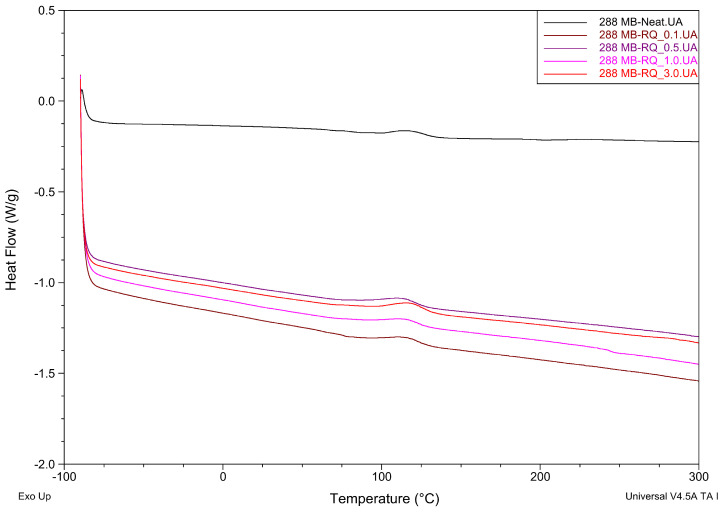
The effect of rose quartz concentration on the maximum temperature and thermal effect of the cross-linking process of 288-MB-RQ adhesive films.

Differential Scanning Calorimetry analysis results of the crosslinking process for three different resins with rose quartz filler are presented in Table 5 and in Figure 7, Figure 8 and Figure 9.

The addition of rose quartz (RQ) filler resulted in minor shifts in the curing temperatures (Ti and Tp). For the Q2-7358 series, a slight decrease in Ti was observed compared to the neat resin, while the Q2-7388 series showed a marginal increase in processing temperatures with higher filler loading. The most apparent shift occurred in the 288 MB series, where the introduction of RQ lowered the onset temperature from 100 °C to 92–94 °C. Such a minor shift shows minor influence on crosslinking kinetics.

Regarding the enthalpy of the reaction (ΔH), the values for all samples remained at a low level (below 9 J/g). While the 288 MB and Q2-7388 series showed a general upward trend in ΔH with increasing filler content, the variations observed in the Q2-7358 series were less consistent. These minor fluctuations in ΔH suggest that while the filler participates in the thermal environment of the matrix, its impact on the overall energetic effect of cross-linking is subtle. The high degree of similarity between the enthalpy values of the neat resins and the composites confirms that the rose quartz acts primarily as a reinforcing phase without fundamentally changing the chemical nature of the siloxane network formation.

### 3.4. Evaluation of Si-PSA-RQ Performance on Different Surfaces

Following key investigations into three Si-PSA compositions containing rose quartz and evaluating the impact of quartz filler on the application properties of the resulting pressure-sensitive adhesives, the composition exhibiting optimal parameters was selected for further testing. It should be noted that in the case of unmodified PSAs, achieving high values for all parameters simultaneously is often unfeasible, as high tack and adhesion typically do not correlate with high cohesion. Therefore, modified compositions are employed—for instance, by incorporating additives to enhance the tack and adhesion of high-cohesion adhesives, or vice versa.

Based on the experimental results, a relatively optimal composition was selected: Q2-7358 supplemented with 0.5 pph of rose quartz. It was demonstrated that the addition of filler substantially reduces shrinkage, confirming its necessity. However, the filler content must be limited, as excessive amounts degrade other performance metrics. The selected composition exhibited robust adhesion (>15% above industrial requirements) and satisfactory tack. At this RQ concentration, cohesion at 70 °C and SAFT (Shear Adhesion Failure Temperature) were superior, exceeding the required standards. Shrinkage after 7 days remained at 0.4%, representing a very favorable result.

**Figure 10:**
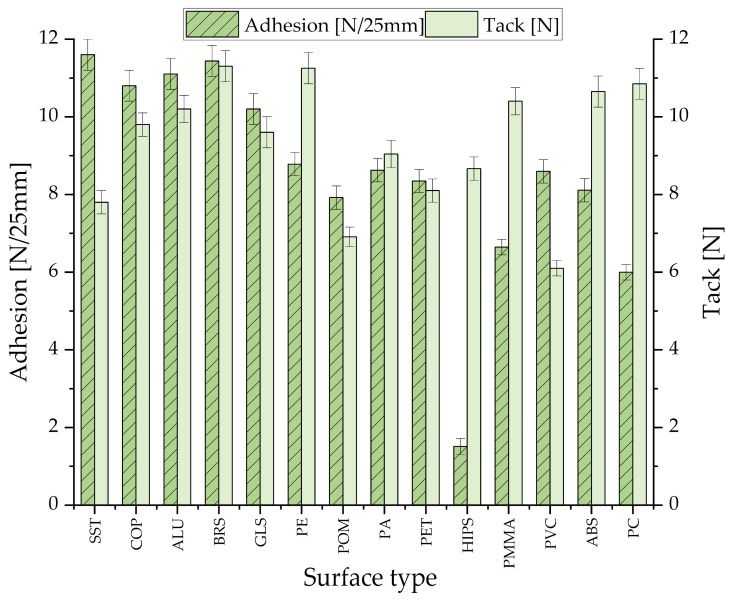
Adhesion and tack results of Q2-7358-05RQ adhesive composition.

The final stage of the research involved testing the selected adhesive composition on surfaces other than the FINAT stainless steel 1.4301 standard (SST) to assess its universality of use in various conditions. The following test substrates were selected: Copper (Cu-ETP H), Aluminum (1050A), Brass (CuZn37), Glass, Polyethylene (PE), Polyacetal (POM), Polyamide PA6 (PA), Polyethylene terephthalate (PET), High-Impact Polystyrene (HIPS), Acrylic glass (PMMA), Polyvinyl chloride unplasticized (PVC), Acrylonitrile butadiene styrene (ABS), and Poly-carbonate (PC) [42,43]. The surfaces were degreased before the adhesive layer was applied. The results of adhesion and tack are presented below in Figure 10, as mean values of three and five samples, respectively (similarly to previous adhesion and tack measurements).

The results reveal that the adhesive composition Q2-7358-05RQ demonstrates robust versatility, maintaining effective bonding performance even on challenging, low-energy surfaces. Substrates such as SST, COP, ALU, BRS, and GLS exhibit high surface energy. The silicone adhesive readily lowers the interfacial energy between the adhesive and the substrate, resulting in robust adhesion. This is evidenced by the consistently high adhesion values (>10 N/25 mm), as the adhesive forms effective chemical and mechanical anchoring points across these surfaces. The effectiveness of the tested adhesive on materials such as PE and POM is noteworthy. Because these polymers have extremely low surface energy, standard organic adhesives often fail to adequately wet the surface [44,45].

Unlike standard organic adhesives, silicone PSAs possess inherently low surface tension, typically around 20–22 mN/m, which allows them to effectively wet substrates with low critical surface tension. According to established adhesion theory, the bonding to non-polar polyolefins is primarily governed by dispersive (van der Waals) interactions at the interface. The high conformational flexibility of the siloxane backbone enables the polymer chains to maximize the effective contact area at the molecular level, facilitating these dispersive forces and potential mechanical interlocking, even in the absence of polar functional groups. This explains the stable adhesion values (8–9 N/25 mm) observed on PE and POM [46]. A clear divergence exists between initial tack (short-term contact) and adhesion (long-term bonding). Materials like PC and PMMA exhibit high tack because the adhesive rapidly wets these smooth, polar-capable surfaces. However, the final adhesion value is lower than on metals, likely due to differences in the chemical interaction (e.g., a lack of strong covalent or hydrogen bonding compared to potential metal–oxide interactions).

A notable deviation was observed for the HIPS substrate, where adhesion dropped to approximately 1.5 N/25 mm despite a moderate initial tack (~8 N). This discrepancy between initial wetting (tack) and long-term bond strength suggests that while the adhesive achieves contact, a stable interface is not established. Further interfacial characterization, such as spectroscopic analysis of the failure mode, would be required to fully elucidate the underlying cause of this specific performance drop.

The cohesion test on inorganic surfaces yielded results identical to those of the SST reference standard, while the SAFT test produced highly comparable results (>200 °C).

## 4. Conclusions

In the search for novel adhesive materials solutions for the energy sector and other industries employing materials operating at elevated temperatures, new Si-PSA compositions incorporating a mineral filler were developed. The addition of rose quartz as a filler to PSA based on polydimethylsiloxane resins resulted in a notable enhancement of performance parameters of the PSAs, including elevated-temperature strength and dimensional stability of the adhesive layer. The resulting compositions also exhibited versatility for application across various structural materials. Optimal results were achieved using the commercial DOWSIL Q2-7358 resin, thermally cross-linked with DClBPO and modified by the addition of 0.5 pph of rose quartz filler. The performance parameters of the adhesive layers derived from it substantially exceeded industrial requirements for properties such as tack, adhesion, shear strength, shrinkage, and elevated-temperature long-term resistance of the joint. The utilized rose quartz is a raw mineral rather than a commercially synthesized filler, prepared solely through mechanical grinding. Its use in this natural state marks a departure from standard industrial practices, offering a novel application route. By limiting preparation to simple cleaning and milling, the process bypasses the financial burden typically associated with chemical modifications or specialized manufacturing. The resulting Si-PSA compositions could serve as an effective alternative to traditional joining methods such as riveting, welding, or structural bonding. They are particularly suited for environments characterized by extreme thermal cycling or sustained high-temperature exposure. Furthermore, the application of these materials as pressure-sensitive adhesives offers a major operational advantage over traditional heat-resistant glues, as they eliminate the need for complex curing cycles or precise mixing ratios. Potential industrial implementations include heavy-duty engine components, exhaust system sealing, and steam turbine assemblies within the heating sector or power engineering.

## Figures and Tables

**Table 1:** Particle size distribution of rose quartz filler.

D_0.5_	D_0.9_	D_3.2_	D_4.3_
[µm]			
8.39 ± 0.74	31.25 ± 0.95	4.28 ± 0.41	19.44 ± 1.85

**Table 2:** The viscosity of various silicone pressure-sensitive adhesives with the addition of 3 pph rose quartz.

Resin	Viscosity (Pa·s)				
	1 Day	2 Days	3 Days	5 Days	7 Days
**Q2-7358**	25.3 ± 0.6	27.1 ± 0.6	32.8 ± 0.8	41.0 ± 0.7	53.0 ± 0.8
**Q2-7388**	12.5 ± 0.5	12.9 ± 0.5	14.8 ± 0.6	17.2 ± 0.6	22.5 ± 0.5
**288 MB**	25.4 ± 0.7	26.3 ± 0.6	27.9 ± 0.7	31.5 ± 0.5	44.0 ± 0.6

**Table 3:** Cohesion and SAFT results for various Si-PSA resins filled with rose quartz.

**Cohesion at 23 °C [h]**	**Q2-7358**	**Q2-7388**	**288-MB**
0.1 pph	>72	>72	>72
0.5 pph	>72	>72	>72
1.0 pph	>72	>72	>72
3.0 pph	>72	>72	>72
**Cohesion at 70 °C [h]**	**Q2-7358**	**Q2-7388**	**288-MB**
0.1 pph	>72	>72	8.3 ± 0.5 af
0.5 pph	>72	>72	17.5 ± 1.2 af/cf
1.0 pph	17.4 ± 1.1 af	18.0 ± 1.4 af	58.0 ± 2.2 af
3.0 pph	10.0 ± 0.6 af	1.5 ± 0.2 af/cf	2.0 ± 0.2 af/cf
**SAFT** **[°C]**	**Q2-7358**	**Q2-7388**	**288-MB**
0.1 pph	>225	>225	>225
0.5 pph	>225	218 ± 6	>225
1.0 pph	217 ± 6	178 ± 4 cf/af	218 ± 5
3.0 pph	216 ± 7	163 ± 5 cf/af	159 ± 5 cf/af

af—adhesion failure; cf—cohesion failure.

**Table 4:** Shrinkage results for various Si-PSA resins filled with rose quartz.

**Shrinkage of Q2-7358**	**0.16** **[h]**	**0.5** **[h]**	**1** **[h]**	**3** **[h]**	**8** **[h]**	**24** **[h]**	**48** **[h]**	**72** **[h]**	**96** **[h]**	**120** **[h]**	**144** **[h]**	**168** **[h]**
0.1 pph	0.14 ± 0.02	0.18 ± 0.02	0.22 ± 0.02	0.28 ± 0.03	0.31 ± 0.02	0.33 ± 0.03	0.35 ± 0.03	0.37 ± 0.02	0.40 ± 0.03	0.43 ± 0.04	0.45 ± 0.04	0.45 ± 0.03
0.5 pph	0.13 ± 0.02	0.16 ± 0.02	0.18 ± 0.02	0.21 ± 0.03	0.24 ± 0.03	0.28 ± 0.03	0.30 ± 0.03	0.33 ± 0.03	0.35 ± 0.03	0.40 ± 0.04	0.40 ± 0.04	0.40 ± 0.04
1.0 pph	0.10 ± 0.01	0.11 ± 0.01	0.12 ± 0.01	0.14 ± 0.02	0.16 ± 0.03	0.17 ± 0.03	0.21 ± 0.04	0.22 ± 0.02	0.28 ± 0.03	0.30 ± 0.03	0.30 ± 0.04	0.30 ± 0.03
3.0 pph	0.08 ± 0.01	0.10 ± 0.01	0.11 ± 0.02	0.13 ± 0.02	0.14 ± 0.03	0.16 ± 0.03	0.17 ± 0.02	0.21 ± 0.03	0.23 ± 0.03	0.24 ± 0.03	0.24 ± 0.03	0.24 ± 0.03
**Shrinkage of Q2-7388**	**0.16** **[h]**	**0.5** **[h]**	**1** **[h]**	**3** **[h]**	**8** **[h]**	**24** **[h]**	**48** **[h]**	**72** **[h]**	**96** **[h]**	**120** **[h]**	**144** **[h]**	**168** **[h]**
0.1 pph	0.15 ± 0.02	0.17 ± 0.02	0.18 ± 0.02	0.20 ± 0.02	0.21 ± 0.03	0.23 ± 0.02	0.25 ± 0.03	0.27 ± 0.03	0.29 ± 0.03	0.30 ± 0.03	0.31 ± 0.03	0.31 ± 0.03
0.5 pph	0.12 ± 0.02	0.13 ± 0.02	0.14 ± 0.02	0.15 ± 0.02	0.17 ± 0.02	0.18 ± 0.02	0.20 ± 0.02	0.23 ± 0.02	0.25 ± 0.03	0.27 ± 0.03	0.27 ± 0.03	0.27 ± 0.03
1.0 pph	0.09 ± 0.01	0.10 ± 0.01	0.11 ± 0.01	0.12 ± 0.02	0.14 ± 0.02	0.15 ± 0.02	0.17 ± 0.02	0.18 ± 0.02	0.20 ± 0.02	0.21 ± 0.02	0.21 ± 0.02	0.21 ± 0.02
3.0 pph	0.08 ± 0.02	0.09 ± 0.01	0.10 ± 0.01	0.10 ± 0.01	0.11 ± 0.01	0.12 ± 0.02	0.13 ± 0.02	0.15 ± 0.02	0.18 ± 0.02	0.18 ± 0.02	0.18 ± 0.02	0.18 ± 0.02
**Shrinkage of 288-MB**	**0.16** **[h]**	**0.5** **[h]**	**1** **[h]**	**3** **[h]**	**8** **[h]**	**24** **[h]**	**48** **[h]**	**72** **[h]**	**96** **[h]**	**120** **[h]**	**144** **[h]**	**168** **[h]**
0.1 pph	0.10 ± 0.01	0.11 ± 0.01	0.13 ± 0.02	0.15 ± 0.02	0.18 ± 0.02	0.20 ± 0.02	0.23 ± 0.02	0.25 ± 0.03	0.28 ± 0.03	0.30 ± 0.03	0.30 ± 0.03	0.30 ± 0.03
0.5 pph	0.09 ± 0.02	0.11 ± 0.01	0.13 ± 0.02	0.15 ± 0.03	0.16 ± 0.02	0.18 ± 0.02	0.21 ± 0.02	0.23 ± 0.03	0.25 ± 0.03	0.27 ± 0.03	0.27 ± 0.03	0.27 ± 0.03
1.0 pph	0.08 ± 0.01	0.10 ± 0.01	0.11 ± 0.02	0.13 ± 0.02	0.14 ± 0.03	0.16 ± 0.02	0.18 ± 0.02	0.18 ± 0.02	0.19 ± 0.02	0.20 ± 0.02	0.22 ± 0.03	0.22 ± 0.03
3.0 pph	0.07 ± 0.02	0.09 ± 0.01	0.10 ± 0.02	0.11 ± 0.01	0.13 ± 0.02	0.15 ± 0.03	0.17 ± 0.02	0.19 ± 0.03	0.20 ± 0.02	0.21 ± 0.02	0.21 ± 0.02	0.21 ± 0.02

**Table 5:** The effect of rose quartz concentration on the maximum temperature and thermal effect of the cross-linking process of the adhesive films.

Sample Name	Ti (°C)	Tp (°C)	ΔH (J/g)
Q2-7358 Neat	101.0 ± 0.7	120.1 ± 0.5	6.0 ± 0.4
Q2-7358 RQ 0.1	97.7 ± 0.6	120.2 ± 0.6	8.5 ± 0.5
Q2-7358 RQ 0.5	99.1 ± 0.5	118.0 ± 0.5	5.9 ± 0.5
Q2-7358 RQ 1.0	98.0 ± 0.7	121.1 ± 0.6	8.5 ± 0.6
Q2-7358 RQ 3.0	99.0 ± 0.6	119.2 ± 0.5	6.4 ± 0.5
Q2-7388 Neat	94.7 ± 0.7	115.0 ± 0.4	5.0 ± 0.4
Q2-7388 RQ 0.1	97.2 ± 0.5	118.0 ± 0.5	6.9 ± 0.6
Q2-7388 RQ 0.5	95.2 ± 0.5	116.1 ± 0.4	4.7 ± 0.4
Q2-7388 RQ 1.0	96.0 ± 0.6	117.0 ± 0.5	7.0 ± 0.5
Q2-7388 RQ 3.0	98.1 ± 0.7	118.1 ± 0.7	7.6 ± 0.6
288 MB Neat	99.9 ± 0.6	116.9 ± 0.6	2.8 ± 0.4
288 MB RQ 0.1	94.1 ± 0.7	115.9 ± 0.3	3.9 ± 0.4
288 MB RQ 0.5	92.0 ± 0.8	113.2 ± 0.7	4.9 ± 0.5
288 MB RQ 1.0	93.9 ± 0.7	113.9 ± 0.4	3.8 ± 0.5
288 MB RQ 3.0	94.0 ± 0.7	116.1 ± 0.5	6.7 ± 0.6

Ti—the onset temperature; Tp—the peak temperature; and ΔH—the enthalpy of the reaction.

## Data Availability

The original contributions presented in this study are included in the article. Further inquiries can be directed to the corresponding authors.

## Abbreviations

The following abbreviations are used in this manuscript:

RQ	Rose quartz
SST	Stainless Steel
COP	Copper
ALU	Aluminum
BRS	Brass
GLS	Glass
PE	Polyethylene
POM	Polyacetal
PA	Polyamide
PET	Poly(ethylene terephthalate)
HIPS	High-Impact Polystyrene
PMMA	Acrylic glass
PVC	Poly(vinyl chloride)
ABS	Poly(acrylonitrile-co-butadiene-co-styrene)
PC	Polycarbonate
